# Reproductive performance of asian catfish ( *Hemibagrus wyckii* Bleeker, 1858), a candidate species for aquaculture

**DOI:** 10.12688/f1000research.14746.2

**Published:** 2018-09-03

**Authors:** Netti Aryani, Indra Suharman, Hafrijal Syandri

**Affiliations:** 1 Department of Aquaculture, Faculty of Fisheries and Marine Science, Universitas Riau, Pekanbaru, 28294, Indonesia; 2 Department of Aquaculture, Faculty of Fisheries and Marine Science, Universitas Bung Hatta, Padang, 25133, Indonesia

**Keywords:** Hemibagrus wyckii, endangered species, alternative fish species, egg quality, sperm

## Abstract

**Background:** *Hemibagrus wyckii* Bagridae is one of the important economic fish species that lives in the rivers and reservoir in Riau Province, Indonesia. The present study aimed to determine the reproductive performance of *H.wyckii* under culture conditions.

**Method** **s**: A total of 10 female and 10 male fish were selected, and weight, length, characteristics of egg and sperm, and hatchery performance were measured. Eggs were fertilized using the dry method. Egg weight and egg diameters were measured for 50 eggs per female. Egg size (50 eggs for each fish) was measured using Labo microscope model  L-711 using software camera 3. Then, saline solution was added over the eggs, followed by the addition of pooled sperm from 10 males.

**Results: **Average relative fecundity, egg weight and egg diameter were 2060±512 eggs/kg fish, 29.86±1.21 mg and 2.67±0.26 mm, respectively. The fertilization rate and hatching rate were 60.91±4.68% and 42.91±2.92% respectively. Sperm characteristics such as volume per fish (mL), pH, concentration (per mL), motility (%) and duration of motility (second) were 0.82±0.20, 7.15±0.12, 3.68±0.15, 72.77±1.46 and 47.5±4.84, respectively.

**Conclusion: **The study results and scientific observations regarding reproductive performance suggest that *H. wyckii* can be considered a new candidate species for aquaculture.

## Introduction

In Indonesia, the fisheries sector plays an important economic role through income generation, diversification of livelihoods, supply of animal proteins, and foreign exchange earnings ^1^. During recent decades, in the freshwater aquaculture sector, the prioritized species for culture were Clarias, Pangasius, Tilapia, Common carp and Giant gourami. Wild fish species in rivers, reservoirs and lakes have not been prioritized for aquaculture operations.

In the Riau Province, there are three rivers, the Kampar Kanan, Kampar Kiri, and Siak rivers, and the Koto Panjang Reservoir. The Kampar Kanan river hosts up to 34 fish species ^2^, the Kampar Kiri river hosts up to 86 fish species ^3^, the Ukai river, a branch of Siak River hosts up to 31 fish species ^4^ and Koto Panjang Reservoir hosts up to 26 fish species ^5^.

*Hemibagrus wyckii* (Bagridae) is one of the most important economic fish species that lives in the rivers and reservoir in Riau Province. *H.wyckii* ( *its* local name is *geso*) is a carnivorous freshwater finfish native to Indonesia ^2, 6^. These fish live in Kampar Kanan and Kampar Kiri rivers with the water temperature between 28 to 29°C, pH 5.20 to 7.20, water transparency 0.50 to 2.30 m, dissolved oxygen 4.87 to 4.97 mg L ^-1^, alkalinity 42.97 to 67.53 mg L ^-1^ and hardness 53.32 to 104.83 mg L ^-1^ ^6^. Furthermore, the average standard lengths of *H.wyckii* from the Koto Panjang Reservoir, Kampar Kanan and Kampar Kiri rivers were 428±15.78, 432.52±66.11 and 425.86±50.41 mm, respectively with the growth model was isometric ^7^. *H. wyckii* has been categorized as of “least concern” by the International Union for Conservation of Nature (IUCN). However, *H.wyckii* in the Kampar Kanan river was categorized as a vulnerable to endangered species ^7, 8^.

Due to the endangered population of *H. wyckii*, it is necessary to domesticate this species as an aquaculture candidate in the future. Therefore, the present study aimed to determine the reproductive performance of *H. wyckii* as a potential species under culture conditions to provide preliminary scientific information and evaluation.

## Methods

### Ethical considerations

While *H. wyckii* is classified as vunerable to endangered in the Kampar Kanan river, the Government of the Republic of Indonesia does not require licenses to obtain, capture and rear this species. Hense, no licenses are applicable to this study. There no suffering animal activity in this study. *H. wyckii* was transported to the pond farm for rearing, injection, ovulation, stripping and sperm production. In the end of the experiment the *H. wyckii* still in good condition until return to the pond.

### Rearing and selection of breeders

Broodfish of *H. wyckii* were collected from upstream areas of the Kampar Kanan river in the Kouk village (0° 19' 23.44˝ N and 100° 56' 40.05˝ E), Kampar Regency, Riau Province. The broodfish kept in oxygenated polythene bag and transported by truck to Sadarlis Green Catfish Farm, Sungai Paku, Kampar Regency, Indonesia. Then, the broodstock of *H. wyckii* had been adapted and reared to maturation under the farm conditions. Prior to stocking, female and male fish were weighed using balance scale (OHAUS model CT 6000-USA), and their lengths were measured using a meter ruler with 0.01mm accuracy. During the grow-out period, fish were cultured in two ponds (4 × 4 × 3 m) separated by sex. The depth of water in each pond was 2.0 m. The inlet of the pond water come from Sungai Paku reservoir at a rate of 2.0 m ^3^ per sec. The broodfish fed with freshwater seashell meat ( *Pilsbryoconcha exilis*; Unionidae) collected from local fisherman near to Sungai Paku Reservoir. The seashell meat was kept at cold box with the temperature 5°C prior given to broodfish. According to Aryani *et al.* ^6^ the proximate composition (% wet weight base) of the seashell meat was 89.37% moisture, 7.08% crude protein, 0.82% fat, 0.29% crude ash and 2.44% carbohydrate. The total of seashell meat given to broodfish everyday were 2.500g per pond (equivalent with 9% body weight of population). The feeding time at 17:00 pm due to carnivorous broodfish. The average weight and length from ten (10) of the female broodfish were 2,669.4±486.917 g and 62.84±8.20 cm, respectively. Meanwhile, the ten (10) of male broodfish were 1,769.1±401.10 g and 54.52±7.17 cm, respectively.

The fish were checked monthly for ovulation and semen production from mid November, 2017 onwards. The broodfish were captured with a gillnet formed into a net bag with the appropriate mesh size and anesthetized by orally with Tricaine methanesulfonate (MS-222, ethyl 4-aminobenzoate methanesulfonate 98%, Sigma Aldrich Co, USA, MO; 50 mg L ^-1^), based on the dosage used for *Solea senegalensis* ^9^. Oocyte maturation was assessed for each individual. The fish were returned to their pond after evaluation, and no mortality occurred. Fish were fasted 48 h prior to the evaluation. Oocytes sampled *in vivo* were taken from females using the method described by Nowosad *et al*. ^10^ and were placed in Serra's solution (6:3:1 of 70% ethanol, 40% formaldehyde and 99.5% acetic acid) for clarification of the cytoplasm. After 5 min, the position of the stage IV oocyte nucleus was determined using criteria by Krejszeff *et al.* ^11^ and was classified as germinal vesicle in the periphery or germinal vesicle breakdown (GVBD).

### Female reproductive performance

*H. wyckii* was categorized as endangered species and difficult to obtain in Kampar Kanan river. A total of 10 mature have been eligible for the experiment. 10 mature females that had oocyte stage IV were sampled from broodstock from 3 ^rd^ week of February to March 2018 in the same farm, and live weights (FeW) and total lengths (FeL) were measured after anesthetization with 0.50 mg L ^-1^ MS-222 ^9^. For ovulation, each female broodfish received two injections of GnRH analogs with a dopamine antagonist (Ovaprim) (manufactured for Syndel Laboratories Ltd, 2595 McCullough Rd. Nanaimo, B.C.V9S 4M9 Canada) applied intraperitoneally under the left pectoral fin. The first injection was 0.2 mL kg BW ^-1^ and the second was 0.6 mL kg BW ^-1^ (total 0.8 mL kg BW ^-1^) at 12 h intervals. These dosages refer to the previous doses for ovulation of *H. wyckii* ^6^. At 18 to 20 h after injection, eggs were stripped into a plastic vessel. Eggs were fertilized using the “dry method” as described by Dabrowski *et al.* ^12^ Egg weights of each female were by determined weighing 50 eggs to the nearest 0.01 g, and egg diameters were measured to the nearest 0.01 mm. Egg size (50 eggs for each fish) was measured using Labo microscope model L-711 using software camera 3. Then, a balanced saline solution (7.5 g of NaCl, 0.2 g of KCl, 0.2 g of CaCl _2_2H _2_O, and 0.02 g of NaHCO _3_, in 1000 mL distilled water) was added over the eggs ^13^, followed by an addition of pooled sperm from 10 males. The eggs were then gently mixed for fertilization and left for three minutes. The fertilized eggs were rinsed several times with incubation water to remove sperm remnants as well as dead and broken eggs. The eggs were left for an additional 25 minutes to facilitate egg hardening by water absorption and disinfected with 100 ppm iodine for 10 minutes. Then, eggs were transferred to incubation trays placed in a vertical hatching system. The water flow rate to each vertical incubator was 3 L minˉ¹. Fifty eggs were randomly sampled at 15 h after fertilization to determine the fertilization rate (FR). The hatching rate (HR) was determined by counting all hatched fry.

### Determination of sperm quality

Males were stimulated with a half-dose of the same hormonal preparations used to stimulate the females. Semen samples were obtained from 10 fish randomly selected from the farm. The male fish were anesthetized with 50 mg Lˉ¹ of MS-222. The doses of the anaesthetic agents were prepared a few minutes before each experiment based on the methods of Weber *et al.* ^9^, and then, weights (MaW) and total lengths (MaL) were measured. Special care was taken to avoid any contamination of semen with urine, feces, mucus and water. Semen samples were collected using plastic syringes in 3 mL aliquots, then placed in an insulated ice-cooled container, transported to the laboratory and analyzed within 2 h.

The sperm evaluation included gross (visual) and microscopic examination (as reviewed by Rurangwa *et al.* ^14^, and Cabrita *et al*. ^15^. The gross examination was based on visual and physical observation of parameters such as the semen volume by collecting the semen in a graduated cylinder and determining the level in milliliters. The microscopic examination was carried out using an Olympus model CX40, with magnification between X 10 and X 25 to determine other parameters such as: motility (duration and percentage). Motility (MO) percentage and duration were determined by observing water activated semen placed on a glass slide under a microscope. Motile sperm were observed and expressed as a percent of non-moving sperm. Motility duration (DMO) was determined as the period between movements of the sperm to cessation of any progressive movement expressed in seconds. Sperm concentration (SC) was measured under a microscope using Neaubeaur’s hemocytometer and calculated as the number of sperm mlˉ¹ ^16^. Semen pH was determined with a hand pH meter (HI8424 Hanna Instruments, USA).

### Water quality

The water temperature of the farm was measured with a thermometer (Celsius scale), and water samples were collected to determine the dissolved oxygen (DO) concentrations. An oxygen meter (YSI model 52, Yellow Spring Instrument Co., Yellow Springs, OH, USA) was used *in situ*, and pH values were determined with a pH meter (Digital Mini-pH Meter, 0-14PH, IQ Scientific, Chemo-science Thailand Co., Ltd, Thailand). Alkalinity and hardness levels of the water were measured according to standard procedures ^17^. The water quality parameters were measured once per month.

### Statistical analysis

Results were given as the means ± SD. Simple linear regression analyses were performed using SPSS software (version 16.0 for Windows; SPSS Inc., Chicago, IL). The standard deviation of each parameter was determined. For linear regression analysis, significant correlations were considered at p<0.05.

## Results

Descriptive measurements and the reproductive performance of female *H.wyckii* are presented in Table 1. Fifty percent of eggs hatched at 60 h (29–30 °C water temperature). The fertilization rate varied between 53.2 and 68.3%, whereas the hatching rate was between 39.5 and 48.3%.

**Table 1.** Female size, egg characteristics and hatchery performance of *Hemibagrus wyckii* (Mean ± SD).

	Variables	Range (Min–Max)
Fish weight (g)	2669.4±486.917	2030 – 3296
Fish length (cm)	62.84±8.20	57.5 – 78.2
Absolute fecundity (egg/fish)	5809±2275	4125 – 9958
Relative fecundity (egg/kg fish)	2060±512	1400 – 3000
Egg diameter (mm)	2.67±0.26	2.10 – 2.86
Hardened egg diameter (mm)	2.94±0.30	2.45 – 2.97
Egg diameter increase (%)	18.27±10.43	4.64 – 32.36
Egg weight (mg)	29.86±1.21	24.4 – 31.8
Hardened egg weight (mg)	30.08±1.20	28.63 – 32.01
Egg weight increase (%)	21.59±1.08	20.02 – 23.5
Fertilization rate (%)	60.91±4.68	53.2 – 68.3
Hatching rate (%)	42.91±2.92	39.5 – 48.3
Hatching weight (mg)	30.08±1.20	28.63 – 32.01

Characteristics of male fish and sperm samples are presented in Table 2. The average live weight of the males is 1,769.1±401.1g. Male *H.wyckii* are found to be slightly smaller than females. In the genital maturation stage, the papilla is not prominent for all male fish as the second sexual characteristic of the other *Hemibagrus*.

**Table 2.** Male size and sperm characteristics of *Hemibagrus wyckii* (Mean ± SD).

	Variables	Range (Min–Max)
Fish weight (g)	1769.1±401.10	1105 – 3127
Fish length (cm)	54.52±7.17	43.7 – 65.2
Gonad weight (g)	29.28±3.42	24.96 – 54.96
Gonadosomatic index (%)	1.95±0.71	1.02 – 2.83
Semen volume (mL)	0.82±0.20	0.60 – 1.20
Semen pH	7.15±0.12	7.30 – 7.70
Sperm concentration (10 ^9^/mL)	3.68±0.15	3.50 – 3.90
Motility (%)	72.77±1.46	70.2 – 75.50
Duration of motility (sec)	47.5±4.84	40.0 – 54.0

According to the analysis of the linear relationship ( *r* ^2^) between variables of *H. wyckii* females shown in Table 3, there was a strong linear relationship between AF and FeW, AF and RF, HEW and EW, HW and EW, HW and HEW, and HR and FR. In contrast, the analysis of the linear relationship ( *r* ^2^) between variables of *H. wyckii* males shown in Table 4, show a strong linear relationship between MaW and MaL, MaW and GW, and MaL and GW.

**Table 3.** Correlations of variables ( *r* ^2^) for *Hemibagrus wyckii* females.

	FeW	FeL	AF	RF	EW	HEW	EWI	ED	HED	HDI	FR	HR	HW
FeL	**0.548**												
AF	**0.812**	**0.687**											
RF	**0.523**	**0.582**	**0.899**										
EW	0.076	0.058	0.127	0.151									
HEW	0.074	0.059	0.126	0.152	**0.999**								
EWI	0.088	0.029	0.056	0.054	0.151	0.148							
ED	0.395	0.381	0.504	0.486	0.464	0.467	0.051						
HED	0.105	0.315	0.196	0.303	**0.572**	**0.579**	0.189	**0.696**					
HDI	0.229	0.598	0.279	0.328	0.220	0.224	0.007	0.234	0.353				
FR	0.087	0.018	0.030	0.003	0.021	0.024	0.001	0.120	0.366	0.091			
HR	0.099	0.026	0.079	0.032	0.043	0.047	0.007	0.133	0.366	0.008	**0.915**		
HW	0.052	0.084	0.112	0.151	**0.974**	**0.976**	0.148	0.467	**0.579**	0.244	0.024	0.031	
GI	0.584	0.665	0.712	0.632	0.471	0.468	0.139	**0.677**	0.438	0.406	0.007	0.020	0.468

Statistically important at *r* ^2^ > 0.500 (underlined) Few: Female fish weight, FeL: Female fish length, AF: Absolute fecundity, RF: Relative fecundity, EW: Egg weight, HEW: Hardened egg weight, EWI: Eggs weight increase, ED: Egg diameter, HED: Hardened egg diameter, HDI: Hardened egg diameter increase, FR: Fertilization rate, HR: Hatching rate, HW: Hatching weight, GI: Gonadosomatic index.

**Table 4.** Correlations of variables ( *r* ^2^) for *Hemibagrus wyckii* males.

	MaW	MaL	GW	GI	MV	pH	SC	MO
MaL	**0.810**							
GW	**0.773**	**0.727**						
GI	0.449	0.496	0.311					
SV	0.049	0.002	0.129	0.066				
pH	0.019	0.047	0.029	0.392	0.204			
SC	0.193	0.110	0.267	0.037	0.001	0.038		
MO	0.184	0.066	0.098	0.004	0.003	0.066	0.133	
DMO	0.121	0.077	0.035	0.015	0.016	0.191	0.139	0.499

Statistically important at *r* ^2^ > 0.500 (underlined) MaW: Male fish weight, MaL: Male fish length, GW: Gonada weight, GI: Gonadosomatic index, MV: Semen volume, SC: Sperm concentration, MO: Motility, DMO: Duration of motility.

The temperature of the pond water ranged from 28°C to 29°C, oxygen ranged from 6.5 mg L ^-1^ to 6.7 mg L ^-1^, pH ranged from 6.5 to 6.8, alkalinity ranged from 42.97 mg L ^-1^ to 57.33 mg L ^-1^ and hardness ranged from 104.83 mg L ^-1 ^to 110.51 mg L ^-1^.

Data of female size, egg characteristic and hatchery performance of Hemibagrus wyckii Click here for additional data file. Copyright: © 2018 Aryani N et al. 2018 Data associated with the article are available under the terms of the Creative Commons Zero "No rights reserved" data waiver (CC0 1.0 Public domain dedication).

## Discussion

In our study, the spawning period of *H. wyckii* started at the 3 ^rd^ week of February and continued until the final examination in March. However, our monthly observations of *H.wyckii* captured by fishermen in the Kampar Kanan river found fish at the gonadal I, II, III and IV stages of development (stage scale by Krejszeff *et al* ^11^), showing a spawning type shows of a partial spawner. Spawning in the wild occurs at the start and end of the rainy season, or this species could spawn twice per year. The spawning type of *H. wyckii* is the same as that of *Hemibagrus nemurus* ^18^. The duration from fertilization to a 50% hatching rate was 60 h with 29°C to 30°C water temperature. The water quality supported the embryonic development of *H.wyckii*. The fertilization rate and hatching rate of various *Hemibagrus* species are reported in Table 5. When findings are compared with other *Hemibagrus* species, the duration of hatching is longer in *H.wyckii*. In other words, *H.wyckii* has species specific characteristics the regarding hatching rate, because the egg diameter was bigger than other *Hemibagrus* eggs ( Table 5).

AFs of *H.wyckii* were between 4125 and 9958 eggs/fish and RFs were between 1400 and 3000 eggs kgˉ¹. Egg production per kg fish (RF) is thought to be more informative than absolute fecundity. RF values of *H.wyckii* were lower compared with those of *H.nemurus* ^19– 21^. In our study, there was no strong linear relationship between RF and fish size. Meanwhile, there was a strong linear relationship between AF and fish size ( Table 3).

**Table 5.** Summary of the egg diameter, fertilization and hatching rate of *H.wyckii*, with respect to those reported for *H.nemurus* and *H.dibrugarensis*.

Species	Eggs diameter (mm)	Fertilization rate (%)	Hatching rate (%)	Reference
*H. wyckii*	2.10 – 2.86	53.2 – 68.3	39.5 – 58.3	This study
*H. wyckii*	2.79 – 2.85	45.8 – 60.2	37.33 – 55.1	6
*H. nemurus*	1.13 – 1.22	74.16 – 77.83	64.16 – 67.83	19
*H. nemurus*	1.07 – 1.21	70.50 – 81.4	63.5 – 71.4	20
*H.dibrugarensis*		34.83 – 77.54	20.61 – 74.32	24
*H.nemurus*	-	93.5 – 94.5	56.91 – 78.3	25
*H. nemurus*	1.57 – 1.75	81.57 – 98.54	81.3 – 96.72	21
*H. nemurus*		39.5 – 79.0	32.5 – 69.7	18

EDs and EWs obtained here ranged between 2.10 and 2.86 mm and 24.4 and 31.8 mg respectively, consistent with those reported by Aryani *et al.* ^6^ At the end of the hardening process, the increases in egg weight and diameter were 29.86 and 14.9% respectively. In the present study, there were strong linear relationships between EW and HEW, and between EW and HW ( Table 3). Lahnsteiner and Patzner ^22^ state that egg weight increases after the hardening process and is linearly correlated with the viability of eggs in Rainbow trout, but not in Alakir trout ^23^.

In this study, the FRs of *H. wyckii* were higher than those of *H.wyckii* from research conducted by Aryani *et al.* ^6^. This suggests that we have improved the method for the fertilization process of sperm and eggs so that hatching rate can increase. Nevertheless, the fertilization rate of *H.wyckii* was lower than that of *H.nemurus* ^17, 24, 25^ ( Table 5). When HR values are compared with other *Hemibagrus* species, HR levels are lower than those of Aryani and Suharman ^14^, Adebiyi *et al.* ^25^ and Suhenda *et al.* ^21^ but higher than those of Aryani *et al.* ^6^ There were strong positive correlations with FR and HR ( *r* ^2^ = 0.91) ( Table 3). Meanwhile, FR and HR were not positively correlated with ED ( Table 3). 

Gonadotropins (GTHs), follicle-stimulating hormone (FSH), luteinizing hormone (LH), and sex steroids are the key regulators of reproduction ^26– 28^. Moreover, numerous circulating endocrine and locally acting paracrine and autocrine factors regulate the various stages of oocyte development and maturation ^29, 30^. Other factors that significantly affect fish eggs are genetic, environmental and stress factors ^31– 33^. However, there is no information about the effects of such kinds of factors on the embryonic development of *H.wyckii*. There are also further information requirements concerning hatchery management of *H.wyckii* such as feed levels and feed type ^6^. During the adaptation period in the present instance, *H. wyckii* were fed with meat freshwater seashell ( *Pilsbryoconcha exilis*; Unionidae), the local name of which is “ *lokan*” which may not be fully suitable for this species, even though this species is carnivorous. The requirement of a balanced feed that meets the nutritional requirements of species being cultured ^34– 36^ and application of a proper feeding program during the ovarian development ^37^ have been emphasized. Suhenda *et al.* ^21^ reported that diet should be offered to *H. nemurus* broodstock at an 8% lipid level in fish feed with 35% crude protein for 4 months to obtain high quality gametes. Meanwhile, Aryani and Suharman ^20^ suggest that a minimum of 32% crude protein should be included in the diet of female *H.nemurus* broodstock. Additionally, implantation of 17ß-estradiol has also managed to improve the reproductive performance of *H.nemurus* ^19^. These issues in *H.wyckii* are yet to be elucidated. Therefore, such research is very important for *H.wyckii* in the future.

Average SVs determined in *H. wyckii* are higher than those in *H.nemurus* (0.10 to 0.35 mL) ^18^ and *Clarias gariepinus* ^38^, but lower than those reported for *Ictalurus punctatus* ^39^. It appears that the semen volume in other species has a positive relationship with sperm concentration, including in *Ictalurus punctatus* ^39^. Meanwhile, in fish farms and hatcheries, the biotic and abiotic factors that affect sperm quality are diverse and dependent on complex interactions between genetic, physiological and environmental factors ^14^. On the other hand, improvements in broodstock nutrition and feeding greatly improve gamete quality and larvae production ^40^.

Semen pH values of *H.wyckii* are consistent with those of other species, including *Barbus grypus* ^41^ and *Carrasius gibelio* ^42^. The sperm motility of *H.wyckii* between 70.2 and 75.50%, and the duration of motility was between 40.0 and 54.0 sec, results that are consistent with *Ictalurus punctatus* ^39^. According to Effer *et al.* ^43^ the duration of sperm motility in fish depends on the temperature of the activation medium. Sperm of *H.wyckii* had an effective fertilizing ability according to the correlation analysis, which did not detect any significant relationship between FR and sperm parameters. However, there was a positive relationship between MO and DMO ( *r* ^2^ = 0.49). Sperm morphology, density, volume, motility and fertilizing capacity, as well as the composition and osmolality of the seminal plasma are parameters commonly measured to assess sperm quality in fish ^14, 44^. In this study, we did not investigate the ionic composition of the semen, but this phenomenon could be related to the ionic composition of semen, which has a significant influence on motility and duration of motility ^45– 47^.

## Conclusion

In conclusion, there was a positive linear relationship between absolute fecundity with body weight and total length. *H.wyckii* reproductive performance under domestication is within the range of data available for aquaculture. Considering that successful larvae productions (and potential juvenile growth) which were possible for *H.wyckii* become an alternative species for aquaculture. However, further studies are clearly required to determine several aspects of this fish under aquaculture conditions.

## Data availability

The data referenced by this article are under copyright with the following copyright statement: Copyright: © 2018 Aryani N et al.

Data associated with the article are available under the terms of the Creative Commons Zero "No rights reserved" data waiver (CC0 1.0 Public domain dedication). 

Dataset 1: Data of female size, egg characteristic and hatchery performance of *Hemibagrus wyckii* 10.5256/f1000research.14746.d204328 ^48^
