# Supplementary material for: Reproductive performance of asian catfish ( Hemibagrus wyckii Bleeker, 1858), a candidate species for aquaculture
Source: F1000Res. 2018 Sep 3;7:683. Originally published 2018 May 31. [Version 2] doi: 10.12688/f1000research.14746.2 (PMC6107977; doi:10.12688/f1000research.14746.2)
Supplement: Data of female size, egg characteristic and hatchery performance of Hemibagrus wyckii [file f1000research-7-17617-s0000.tgz › 50b9a531-1e62-43de-a743-8485209b779e_Raw_Data_of_Hemibagrus_wyckii_for_manuscript_No._14746,_27_Apr_'18.docx]

Tabel 1. The data of fish length. weight.absolute fecundity and relative fecundity of Hemibagrus wyckii

| **NO** | **Fish length (cm)** | **Fish weight (g)** | **Absolute fecundity (egg/fish)** | **Relative fecundity (egg/kg fish)** |
| --- | --- | --- | --- | --- |
| 1 | 57.50 | 2030 | 4125 | 2000 |
| 2 | 58.30 | 2120 | 3684 | 1700 |
| 3 | 62.10 | 2784 | 7210 | 2500 |
| 4 | 76.60 | 3200 | 7338 | 2200 |
| 5 | 55.20 | 2246 | 3627 | 1600 |
| 6 | 62.26 | 3296 | 7970 | 2400 |
| 7 | 58.00 | 2472 | 3910 | 1500 |
| 8 | 56.00 | 2319 | 3412 | 1400 |
| 9 | 64.30 | 3127 | 7265 | 2300 |
| 10 | 78.20 | 3100 | 9558 | 3000 |

Tabel 2. Egg diameter of *Hemibagrus wyckii*

|  | **Number of fish** | | | | | | | | | |
| --- | --- | --- | --- | --- | --- | --- | --- | --- | --- | --- |
| **No** | **1** | **2** | **3** | **4** | **5** | **6** | **7** | **8** | **9** | **10** |
| 1 | 2.60 | 2.80 | 2.86 | 2.85 | 2.12 | 2.85 | 2.60 | 2.35 | 2.82 | 2.84 |
| 2 | 2.62 | 2.80 | 2.86 | 2.85 | 2.11 | 2.85 | 2.60 | 2.35 | 2.82 | 2.83 |
| 3 | 2.58 | 2.79 | 2.85 | 2.85 | 2.13 | 2.86 | 2.60 | 2.35 | 2.81 | 2.84 |
| 4 | 2.60 | 2.81 | 2.85 | 2.86 | 2.13 | 2.86 | 2.61 | 2.35 | 2.81 | 2.83 |
| 5 | 2.59 | 2.80 | 2.86 | 2.86 | 2.12 | 2.85 | 2.61 | 2.36 | 2.83 | 2.83 |
| 6 | 2.61 | 2.79 | 2.87 | 2.86 | 2.11 | 2.85 | 2.61 | 2.36 | 2.82 | 2.83 |
| 7 | 2.61 | 2.81 | 2.87 | 2.86 | 2.11 | 2.84 | 2.61 | 2.34 | 2.82 | 2.81 |
| 8 | 2.59 | 2.78 | 2.86 | 2.87 | 2.12 | 2.84 | 2.59 | 2.34 | 2.81 | 2.81 |
| 9 | 2.60 | 2.82 | 2.87 | 2.87 | 2.12 | 2.85 | 2.59 | 2.34 | 2.81 | 2.83 |
| 10 | 2.60 | 2.80 | 2.87 | 2.87 | 2.19 | 2.86 | 2.59 | 2.34 | 2.83 | 2.82 |
| 11 | 2.58 | 2.79 | 2.85 | 2.87 | 2.21 | 2.86 | 2.61 | 2.36 | 2.83 | 2.83 |
| 12 | 2.62 | 2.79 | 2.85 | 2.85 | 2.21 | 2.85 | 2.60 | 2.35 | 2.82 | 2.83 |
| 13 | 2.60 | 2.80 | 2.85 | 2.84 | 2.21 | 2.84 | 2.61 | 2.36 | 2.82 | 2.84 |
| 14 | 2.62 | 2.85 | 2.86 | 2.85 | 2.21 | 2.86 | 2.59 | 2.35 | 2.82 | 2.84 |
| 15 | 2.58 | 2.85 | 2.86 | 2.88 | 2.19 | 2.84 | 2.60 | 2.36 | 2.82 | 2.85 |
| 16 | 2.60 | 2.81 | 2.86 | 2.87 | 2.19 | 2.85 | 2.60 | 2.34 | 2.83 | 2.85 |
| 17 | 2.59 | 2.84 | 2.85 | 2.86 | 2.19 | 2.85 | 2.60 | 2.34 | 2.82 | 2.85 |
| 18 | 2.61 | 2.84 | 2.84 | 2.85 | 2.12 | 2.85 | 2.59 | 2.35 | 2.81 | 2.84 |
| 19 | 2.61 | 2.82 | 2.85 | 2.85 | 2.21 | 2.85 | 2.59 | 2.35 | 2.80 | 2.84 |
| 20 | 2.59 | 2.81 | 2.88 | 2.86 | 2.11 | 2.84 | 2.59 | 2.36 | 2.81 | 2.84 |
| 21 | 2.60 | 2.79 | 2.87 | 2.87 | 2.12 | 2.84 | 2.61 | 2.36 | 2.82 | 2.83 |
| 22 | 2.60 | 2.81 | 2.86 | 2.85 | 2.12 | 2.85 | 2.61 | 2.35 | 2.82 | 2.83 |
| 23 | 2.58 | 2.79 | 2.85 | 2.85 | 2.18 | 2.86 | 2.59 | 2.35 | 2.81 | 2.85 |
| 24 | 2.62 | 2.79 | 2.85 | 2.85 | 2.12 | 2.85 | 2.61 | 2.35 | 2.82 | 2.85 |
| 25 | 2.58 | 2.80 | 2.86 | 2.86 | 2.19 | 2.85 | 2.6 | 2.35 | 2.83 | 2.84 |
| 26 | 2.58 | 2.80 | 2.87 | 2.86 | 2.12 | 2.84 | 2.59 | 2.36 | 2.81 | 2.84 |
| 27 | 2.59 | 2.81 | 2.85 | 2.86 | 2.11 | 2.85 | 2.60 | 2.35 | 2.81 | 2.84 |
| 28 | 2.59 | 2.81 | 2.84 | 2.85 | 2.11 | 2.86 | 2.60 | 2.35 | 2.81 | 2.83 |
| 29 | 2.60 | 2.81 | 2.88 | 2.85 | 2.12 | 2.86 | 2.61 | 2.35 | 2.81 | 2.84 |
| 30 | 2.60 | 2.79 | 2.87 | 2.85 | 2.13 | 2.86 | 2.61 | 2.36 | 2.8 | 2.85 |
| 31 | 2.60 | 2.85 | 2.85 | 2.86 | 2.13 | 2.84 | 2.59 | 2.36 | 2.8 | 2.85 |
| 32 | 2.60 | 2.81 | 2.86 | 2.86 | 2.11 | 2.85 | 2.60 | 2.34 | 2.83 | 2.84 |
| 33 | 2.61 | 2.78 | 2.86 | 2.86 | 2.11 | 2.85 | 2.60 | 2.34 | 2.81 | 2.85 |
| 34 | 2.61 | 2.82 | 2.85 | 2.86 | 2.12 | 2.85 | 2.59 | 2.34 | 2.81 | 2.85 |
| 35 | 2.62 | 2.82 | 2.85 | 2.87 | 2.12 | 2.85 | 2.60 | 2.34 | 2.80 | 2.84 |
| 36 | 2.62 | 2.79 | 2.84 | 2.87 | 2.12 | 2.85 | 2.61 | 2.34 | 2.81 | 2.84 |
| 37 | 2.63 | 2.81 | 2.87 | 2.87 | 2.12 | 2.84 | 2.61 | 2.34 | 2.82 | 2.84 |
| 38 | 2.57 | 2.81 | 2.86 | 2.87 | 2.12 | 2.86 | 2.61 | 2.35 | 2.82 | 2.84 |
| 39 | 2.60 | 2.86 | 2.88 | 2.84 | 2.11 | 2.86 | 2.59 | 2.35 | 2.81 | 2.85 |
| 40 | 2.59 | 2.80 | 2.87 | 2.88 | 2.11 | 2.85 | 2.59 | 2.36 | 2.81 | 2.84 |
| 41 | 2.61 | 2.85 | 2.86 | 2.87 | 2.11 | 2.84 | 2.60 | 2.36 | 2.82 | 2.84 |
| 42 | 2.62 | 2.81 | 2.85 | 2.85 | 2.12 | 2.85 | 2.60 | 2.36 | 2.82 | 2.84 |
| 43 | 2.59 | 2.81 | 2.85 | 2.86 | 2.12 | 2.86 | 2.61 | 2.36 | 2.81 | 2.83 |
| 44 | 2.58 | 2.79 | 2.85 | 2.86 | 2.12 | 2.85 | 2.61 | 2.36 | 2.82 | 2.84 |
| 45 | 2.61 | 2.79 | 2.87 | 2.85 | 2.11 | 2.84 | 2.61 | 2.35 | 2.82 | 2.85 |
| 46 | 2.59 | 2.81 | 2.87 | 2.85 | 2.11 | 2.84 | 2.60 | 2.36 | 2.81 | 2.85 |
| 47 | 2.60 | 2.85 | 2.87 | 2.84 | 2.11 | 2.85 | 2.60 | 2.34 | 2.82 | 2.85 |
| 48 | 2.61 | 2.80 | 2.86 | 2.87 | 2.12 | 2.85 | 2.60 | 2.34 | 2.82 | 2.84 |
| 49 | 2.58 | 2.79 | 2.86 | 2.86 | 2.11 | 2.84 | 2.59 | 2.35 | 2.81 | 2.84 |
| 50 | 2.62 | 2.81 | 2.86 | 2.86 | 2.12 | 2.86 | 2.60 | 2.35 | 2.81 | 2.84 |

Tabel 3. The data of Hardened egg diameter (mm) of *Hemibagrus wyckii*

|  | **Number of Fish** | | | | | | | | |
| --- | --- | --- | --- | --- | --- | --- | --- | --- | --- |
| **No** | **1** | **2** | **3** | **4** | **5** | **6** | **7** | **8** | **9** |
| 1 | 2.86 | 2.85 | 2.95 | 2.94 | 2.45 | 2.97 | 2.93 | 2.67 | 2.95 |
| 2 | 2.86 | 2.87 | 2.94 | 2.96 | 2.46 | 2.96 | 2.93 | 2.67 | 2.96 |
| 3 | 2.86 | 2.86 | 2.95 | 2.95 | 2.46 | 2.97 | 2.92 | 2.68 | 2.97 |
| 4 | 2.86 | 2.86 | 2.96 | 2.95 | 2.45 | 2.98 | 2.93 | 2.66 | 2.94 |
| 5 | 2.86 | 2.84 | 2.94 | 2.95 | 2.43 | 2.95 | 2.94 | 2.67 | 2.95 |
| 6 | 2.87 | 2.85 | 2.95 | 2.94 | 2.45 | 2.96 | 2.94 | 2.65 | 2.95 |
| 7 | 2.87 | 2.85 | 2.95 | 2.94 | 2.44 | 2.97 | 2.92 | 2.65 | 2.96 |
| 8 | 2.88 | 2.84 | 2.94 | 2.94 | 2.44 | 2.98 | 2.91 | 2.66 | 2.94 |
| 9 | 2.88 | 2.85 | 2.96 | 2.95 | 2.45 | 2.98 | 2.92 | 2.67 | 2.96 |
| 10 | 2.87 | 2.86 | 2.95 | 2.95 | 2.44 | 2.97 | 2.91 | 2.68 | 2.95 |
| 11 | 2.87 | 2.85 | 2.94 | 2.94 | 2.45 | 2.97 | 2.94 | 2.67 | 2.95 |
| 12 | 2.85 | 2.85 | 2.95 | 2.94 | 2.46 | 2.96 | 2.93 | 2.68 | 2.96 |
| 13 | 2.84 | 2.86 | 2.95 | 2.96 | 2.45 | 2.96 | 2.93 | 2.67 | 2.97 |
| 14 | 2.86 | 2.87 | 2.96 | 2.96 | 2.46 | 2.96 | 2.92 | 2.67 | 2.96 |
| 15 | 2.88 | 2.85 | 2.95 | 2.94 | 2.46 | 2.96 | 2.92 | 2.68 | 2.96 |
| 16 | 2.85 | 2.85 | 2.95 | 2.95 | 2.44 | 2.96 | 2.91 | 2.67 | 2.96 |
| 17 | 2.86 | 2.87 | 2.95 | 2.94 | 2.44 | 2.97 | 2.92 | 2.67 | 2.96 |
| 18 | 2.87 | 2.86 | 2.94 | 2.95 | 2.45 | 2.97 | 2.91 | 2.68 | 2.96 |
| 19 | 2.88 | 2.85 | 2.96 | 2.95 | 2.45 | 2.98 | 2.91 | 2.68 | 2.94 |
| 20 | 2.88 | 2.86 | 2.96 | 2.96 | 2.46 | 2.98 | 2.93 | 2.68 | 2.96 |
| 21 | 2.87 | 2.85 | 2.95 | 2.96 | 2.46 | 2.97 | 2.93 | 2.67 | 2.95 |
| 22 | 2.87 | 2.85 | 2.94 | 2.94 | 2.45 | 2.97 | 2.93 | 2.68 | 2.94 |
| 23 | 2.86 | 2.84 | 2.95 | 2.94 | 2.45 | 2.98 | 2.94 | 2.67 | 2.94 |
| 24 | 2.86 | 2.85 | 2.95 | 2.95 | 2.45 | 2.96 | 2.93 | 2.68 | 2.95 |
| 25 | 2.86 | 2.85 | 2.94 | 2.95 | 2.46 | 2.97 | 2.92 | 2.67 | 2.96 |
| 26 | 2.86 | 2.86 | 2.95 | 2.95 | 2.45 | 2.98 | 2.93 | 2.68 | 2.94 |
| 27 | 2.86 | 2.84 | 2.95 | 2.96 | 2.45 | 2.96 | 2.94 | 2.67 | 2.96 |
| 28 | 2.87 | 2.85 | 2.95 | 2.96 | 2.45 | 2.96 | 2.91 | 2.67 | 2.95 |
| 29 | 2.87 | 2.85 | 2.95 | 2.95 | 2.45 | 2.97 | 2.91 | 2.68 | 2.96 |
| 30 | 2.87 | 2.86 | 2.95 | 2.94 | 2.44 | 2.94 | 2.92 | 2.68 | 2.95 |
| 31 | 2.84 | 2.89 | 2.94 | 2.95 | 2.44 | 2.96 | 2.92 | 2.67 | 2.96 |
| 32 | 2.86 | 2.85 | 2.95 | 2.95 | 2.45 | 2.97 | 2.91 | 2.67 | 2.95 |
| 33 | 2.87 | 2.85 | 2.96 | 2.96 | 2.46 | 2.97 | 2.92 | 2.67 | 2.96 |
| 34 | 2.87 | 2.86 | 2.96 | 2.95 | 2.44 | 2.95 | 2.92 | 2.67 | 2.96 |
| 35 | 2.85 | 2.86 | 2.95 | 2.95 | 2.45 | 2.96 | 2.93 | 2.68 | 2.95 |
| 36 | 2.86 | 2.86 | 2.95 | 2.96 | 2.45 | 2.97 | 2.93 | 2.65 | 2.97 |
| 37 | 2.87 | 2.87 | 2.96 | 2.96 | 2.46 | 2.98 | 2.91 | 2.66 | 2.96 |
| 38 | 2.88 | 2.87 | 2.96 | 2.95 | 2.45 | 2.97 | 2.91 | 2.57 | 2.95 |
| 39 | 2.87 | 2.89 | 2.95 | 2.94 | 2.45 | 2.98 | 2.93 | 2.67 | 2.96 |
| 40 | 2.88 | 2.86 | 2.94 | 2.94 | 2.45 | 2.97 | 2.92 | 2.68 | 2.96 |
| 41 | 2.87 | 2.86 | 2.94 | 2.94 | 2.45 | 2.98 | 2.93 | 2.67 | 2.95 |
| 42 | 2.88 | 2.85 | 2.95 | 2.95 | 2.45 | 2.97 | 2.93 | 2.66 | 2.95 |
| 43 | 2.87 | 2.85 | 2.96 | 2.95 | 2.45 | 2.94 | 2.94 | 2.67 | 2.96 |
| 44 | 2.84 | 2.84 | 2.95 | 2.96 | 2.45 | 2.97 | 2.94 | 2.67 | 2.94 |
| 45 | 2.87 | 2.85 | 2.96 | 2.96 | 2.46 | 2.96 | 2.93 | 2.68 | 2.94 |
| 46 | 2.85 | 2.84 | 2.95 | 2.95 | 2.45 | 2.97 | 2.93 | 2.66 | 2.95 |
| 47 | 2.86 | 2.89 | 2.94 | 2.94 | 2.45 | 2.94 | 2.92 | 2.68 | 2.95 |
| 48 | 2.87 | 2.85 | 2.94 | 2.95 | 2.45 | 2.97 | 2.92 | 2.67 | 2.95 |
| 49 | 2.87 | 2.85 | 2.95 | 2.95 | 2.45 | 2.97 | 2.91 | 2.68 | 2.94 |
| 50 | 2.87 | 2.84 | 2.94 | 2.95 | 2.45 | 2.97 | 2.93 | 2.67 | 2.96 |

Tabel 4. The data of Egg diameter increase (%) of *Hemibagrus wyckii*

|  | **Number of Fish** | | | | | | | | | |
| --- | --- | --- | --- | --- | --- | --- | --- | --- | --- | --- |
| **No** | **1** | **2** | **3** | **4** | **5** | **6** | **7** | **8** | **9** | **10** |
| 1 | 26 | 5 | 9 | 9 | 33 | 12 | 33 | 32 | 13 | 12 |
| 2 | 24 | 7 | 8 | 11 | 35 | 11 | 33 | 32 | 14 | 13 |
| 3 | 28 | 7 | 10 | 10 | 33 | 11 | 32 | 33 | 16 | 12 |
| 4 | 26 | 5 | 11 | 9 | 32 | 12 | 32 | 31 | 13 | 13 |
| 5 | 27 | 4 | 8 | 9 | 31 | 10 | 33 | 31 | 12 | 13 |
| 6 | 26 | 6 | 8 | 8 | 34 | 11 | 33 | 29 | 13 | 14 |
| 7 | 26 | 4 | 8 | 8 | 33 | 13 | 31 | 31 | 14 | 16 |
| 8 | 29 | 6 | 8 | 7 | 32 | 14 | 32 | 32 | 13 | 17 |
| 10 | 28 | 3 | 9 | 8 | 33 | 13 | 33 | 33 | 15 | 15 |
| 11 | 27 | 6 | 8 | 8 | 25 | 11 | 32 | 34 | 12 | 15 |
| 12 | 29 | 6 | 9 | 7 | 24 | 11 | 33 | 31 | 12 | 14 |
| 12 | 23 | 6 | 10 | 9 | 25 | 11 | 33 | 33 | 14 | 12 |
| 13 | 24 | 6 | 10 | 12 | 24 | 12 | 32 | 31 | 15 | 10 |
| 14 | 24 | 2 | 10 | 11 | 25 | 10 | 33 | 32 | 14 | 12 |
| 15 | 30 | 0 | 9 | 6 | 27 | 12 | 32 | 32 | 14 | 13 |
| 16 | 25 | 4 | 9 | 8 | 25 | 11 | 31 | 33 | 13 | 10 |
| 17 | 27 | 3 | 10 | 8 | 25 | 12 | 32 | 33 | 14 | 11 |
| 18 | 26 | 2 | 10 | 10 | 33 | 12 | 32 | 33 | 15 | 13 |
| 19 | 27 | 3 | 11 | 10 | 24 | 13 | 32 | 33 | 14 | 14 |
| 20 | 29 | 5 | 8 | 10 | 35 | 14 | 34 | 32 | 15 | 14 |
| 21 | 27 | 6 | 8 | 9 | 34 | 13 | 32 | 31 | 13 | 14 |
| 22 | 27 | 4 | 8 | 9 | 33 | 12 | 32 | 33 | 12 | 14 |
| 23 | 28 | 5 | 10 | 9 | 27 | 12 | 35 | 32 | 13 | 11 |
| 24 | 24 | 6 | 10 | 10 | 33 | 11 | 32 | 33 | 13 | 11 |
| 25 | 28 | 5 | 8 | 9 | 27 | 12 | 32 | 32 | 13 | 12 |
| 26 | 28 | 6 | 8 | 9 | 33 | 14 | 34 | 32 | 13 | 12 |
| 27 | 27 | 3 | 10 | 10 | 34 | 11 | 34 | 32 | 15 | 12 |
| 28 | 28 | 4 | 11 | 11 | 34 | 10 | 31 | 32 | 14 | 14 |
| 29 | 27 | 4 | 7 | 10 | 33 | 11 | 30 | 33 | 15 | 13 |
| 30 | 27 | 7 | 8 | 9 | 31 | 8 | 31 | 32 | 15 | 12 |
| 31 | 24 | 4 | 9 | 9 | 31 | 12 | 33 | 31 | 16 | 9 |
| 32 | 26 | 4 | 9 | 9 | 34 | 12 | 31 | 33 | 12 | 12 |
| 33 | 26 | 7 | 10 | 10 | 35 | 12 | 32 | 33 | 15 | 12 |
| 34 | 26 | 4 | 11 | 9 | 32 | 10 | 33 | 33 | 15 | 12 |
| 35 | 23 | 4 | 10 | 8 | 33 | 11 | 33 | 34 | 15 | 11 |
| 36 | 24 | 7 | 11 | 9 | 33 | 12 | 32 | 31 | 16 | 12 |
| 37 | 24 | 6 | 9 | 9 | 34 | 14 | 30 | 32 | 14 | 13 |
| 38 | 31 | 6 | 10 | 8 | 33 | 11 | 30 | 22 | 13 | 14 |
| 39 | 27 | 3 | 7 | 10 | 34 | 12 | 34 | 32 | 15 | 12 |
| 40 | 29 | 6 | 7 | 6 | 34 | 12 | 33 | 32 | 15 | 14 |
| 41 | 26 | 1 | 8 | 7 | 34 | 14 | 33 | 31 | 13 | 13 |
| 42 | 26 | 4 | 10 | 10 | 33 | 12 | 33 | 30 | 13 | 14 |
| 43 | 28 | 4 | 11 | 9 | 33 | 8 | 33 | 31 | 15 | 14 |
| 44 | 26 | 5 | 10 | 10 | 33 | 12 | 33 | 31 | 12 | 10 |
| 46 | 26 | 6 | 9 | 11 | 35 | 12 | 32 | 33 | 12 | 12 |
| 46 | 26 | 3 | 8 | 10 | 34 | 13 | 33 | 30 | 14 | 10 |
| 47 | 26 | 4 | 7 | 10 | 34 | 9 | 32 | 34 | 13 | 11 |
| 48 | 26 | 5 | 8 | 8 | 33 | 12 | 32 | 33 | 13 | 13 |
| 49 | 29 | 6 | 9 | 9 | 34 | 13 | 32 | 33 | 13 | 13 |
| 50 | 25 | 3 | 8 | 9 | 33 | 11 | 33 | 32 | 15 | 13 |

Tabel 5. The data of egg weight (mg) of *Hemibagrus wyckii*

|  | **Number of Fish** | | | | | | | | | |
| --- | --- | --- | --- | --- | --- | --- | --- | --- | --- | --- |
| **No** | **1** | **2** | **3** | **4** | **5** | **6** | **7** | **8** | **9** | **10** |
| 1 | 28.9 | 31.7 | 31.6 | 30.5 | 28.8 | 30.7 | 28.8 | 28.4 | 29.5 | 29.6 |
| 2 | 29.0 | 31.9 | 31.6 | 30.6 | 28.8 | 30.6 | 28.8 | 28.4 | 29.6 | 29.7 |
| 3 | 28.8 | 31.8 | 31.6 | 30.5 | 28.9 | 30.7 | 28.9 | 28.5 | 29.4 | 29.6 |
| 4 | 29.0 | 31.8 | 31.5 | 30.6 | 28.8 | 30.7 | 28.7 | 28.4 | 29.5 | 29.7 |
| 5 | 29.3 | 31.9 | 31.6 | 30.4 | 28.9 | 30.6 | 28.8 | 28.5 | 29.5 | 29.8 |
| 6 | 29.3 | 31.7 | 31.5 | 30.4 | 28.8 | 30.6 | 28.8 | 28.4 | 29.5 | 29.6 |
| 7 | 29.5 | 31.8 | 31.4 | 30.5 | 28.9 | 30.8 | 28.9 | 28.4 | 29.4 | 29.6 |
| 8 | 29.7 | 31.9 | 31.5 | 30.5 | 28.8 | 30.7 | 28.8 | 28.4 | 29.5 | 29.5 |
| 9 | 29.9 | 31.8 | 31.5 | 30.6 | 28.8 | 30.6 | 28.9 | 28.4 | 29.6 | 29.5 |
| 10 | 28.5 | 31.7 | 31.6 | 30.5 | 28.9 | 30.8 | 28.8 | 28.5 | 29.6 | 29.5 |
| 11 | 28.9 | 31.8 | 31.5 | 30.5 | 28.8 | 30.6 | 28.8 | 28.4 | 29.5 | 29.6 |
| 12 | 28.7 | 31.9 | 31.4 | 30.5 | 28.9 | 30.6 | 28.8 | 28.5 | 29.5 | 29.6 |
| 13 | 28.8 | 31.8 | 31.6 | 30.6 | 28.9 | 30.7 | 28.8 | 28.4 | 29.5 | 29.6 |
| 14 | 28.9 | 31.7 | 31.5 | 30.5 | 28.8 | 30.7 | 28.7 | 28.4 | 29.4 | 29.6 |
| 15 | 29.2 | 31.9 | 31.5 | 30.6 | 28.8 | 30.7 | 28.8 | 28.4 | 29.4 | 29.7 |
| 16 | 29.1 | 31.9 | 31.5 | 30.5 | 28.8 | 30.6 | 28.8 | 28.5 | 29.4 | 29.5 |
| 17 | 28.7 | 31.9 | 31.6 | 30.4 | 28.8 | 30.8 | 28.9 | 28.6 | 29.4 | 29.6 |
| 18 | 28.8 | 31.9 | 31.5 | 30.4 | 28.8 | 30.7 | 28.8 | 28.5 | 29.5 | 29.7 |
| 19 | 28.9 | 31.8 | 31.6 | 30.5 | 28.9 | 30.6 | 28.8 | 28.4 | 29.5 | 29.6 |
| 20 | 28.7 | 31.8 | 31.5 | 30.5 | 28.9 | 30.8 | 28.8 | 28.4 | 29.5 | 29.6 |
| 21 | 28.9 | 31.8 | 31.4 | 30.5 | 28.8 | 30.7 | 28.9 | 28.5 | 29.5 | 29.6 |
| 22 | 28.6 | 31.9 | 31.5 | 30.5 | 28.9 | 30.6 | 28.7 | 28.6 | 29.4 | 29.7 |
| 23 | 28.9 | 31.9 | 31.5 | 30.5 | 28.8 | 30.7 | 28.7 | 28.4 | 29.5 | 29.6 |
| 24 | 29.1 | 31.7 | 31.5 | 30.6 | 28.9 | 30.7 | 28.7 | 28.5 | 29.5 | 29.7 |
| 25 | 29.0 | 31.8 | 31.6 | 30.6 | 28.7 | 30.7 | 28.8 | 28.5 | 29.6 | 29.6 |
| 26 | 28.7 | 31.9 | 31.6 | 30.6 | 28.8 | 30.6 | 28.8 | 28.5 | 29.6 | 29.7 |
| 27 | 28.9 | 31.9 | 31.5 | 30.5 | 28.9 | 30.8 | 28.9 | 28.4 | 29.5 | 29.7 |
| 28 | 28.9 | 31.8 | 31.5 | 30.5 | 28.8 | 30.7 | 28.8 | 28.5 | 29.5 | 29.7 |
| 29 | 29.8 | 31.8 | 31.5 | 30.5 | 28.8 | 30.8 | 28.8 | 28.5 | 29.5 | 29.6 |
| 30 | 28.9 | 31.8 | 31.5 | 30.6 | 28.9 | 30.7 | 28.8 | 28.4 | 29.5 | 29.7 |
| 31 | 28.7 | 31.9 | 31.5 | 30.6 | 28.8 | 30.7 | 28.7 | 28.6 | 29.5 | 29.7 |
| 32 | 28.9 | 31.9 | 31.4 | 30.6 | 28.9 | 30.8 | 28.7 | 28.5 | 29.5 | 29.6 |
| 33 | 29.1 | 31.7 | 31.6 | 30.5 | 28.8 | 30.6 | 28.8 | 28.4 | 29.6 | 29.6 |
| 34 | 28.7 | 31.9 | 31.6 | 30.5 | 28.8 | 30.7 | 28.9 | 28.4 | 29.6 | 29.5 |
| 35 | 28.8 | 31.8 | 31.5 | 30.5 | 28.9 | 30.66 | 28.8 | 28.5 | 29.6 | 29.6 |
| 36 | 28.9 | 31.1 | 31.5 | 30.6 | 28.8 | 30.8 | 28.8 | 28.4 | 29.4 | 29.6 |
| 37 | 29.0 | 31.8 | 31.5 | 30.5 | 28.9 | 30.7 | 28.9 | 28.4 | 29.3 | 29.6 |
| 38 | 29.0 | 31.9 | 31.6 | 30.5 | 28.8 | 30.8 | 28.7 | 28.4 | 29.4 | 29.6 |
| 39 | 28.9 | 31.8 | 31.6 | 30.5 | 28.9 | 30.8 | 28.7 | 28.5 | 29.4 | 29.7 |
| 40 | 28.7 | 31.9 | 31.6 | 30.6 | 28.9 | 30.7 | 28.7 | 28.4 | 29.3 | 29.7 |
| 41 | 28.9 | 31.8 | 31.5 | 30.6 | 28.9 | 30.7 | 28.8 | 28.4 | 29.5 | 29.7 |
| 42 | 28.8 | 31.8 | 31.5 | 30.6 | 28.8 | 30.7 | 28.8 | 28.4 | 29.5 | 29.6 |
| 43 | 28.9 | 31.8 | 31.5 | 30.5 | 28.9 | 30.8 | 28.9 | 28.5 | 29.5 | 29.6 |
| 44 | 29.0 | 31.9 | 31.6 | 30.5 | 28.9 | 30.7 | 28.7 | 28.4 | 29.5 | 29.7 |
| 45 | 28.8 | 31.8 | 31.5 | 30.4 | 28.8 | 30.7 | 28.9 | 28.4 | 29.5 | 29.6 |
| 46 | 28.7 | 31.8 | 31.5 | 30.5 | 28.8 | 30.7 | 28.8 | 28.5 | 29.5 | 29.7 |
| 47 | 29.0 | 31.9 | 31.4 | 30.5 | 28.9 | 30.6 | 28.9 | 28.4 | 29.6 | 29.6 |
| 48 | 28.9 | 31.8 | 31.5 | 30.6 | 28.8 | 30.8 | 29 | 28.5 | 29.6 | 29.6 |
| 49 | 28.8 | 31.8 | 31.5 | 30.5 | 28.9 | 30.6 | 28.8 | 28.4 | 29.3 | 29.7 |
| 50 | 28.9 | 31.9 | 31.6 | 30.6 | 28.8 | 30.7 | 28.7 | 28.4 | 29.4 | 29.6 |

Tabel 6. The data of hardened egg weight (mg) of *Hemibagrus wyckii*

|  | **Number of fish** | | | | | | | | | |
| --- | --- | --- | --- | --- | --- | --- | --- | --- | --- | --- |
| **No** | **1** | **2** | **3** | **4** | **5** | **6** | **7** | **8** | **9** | **10** |
| 1 | 29.1 | 32 | 31.8 | 30.8 | 29 | 30.9 | 29 | 28.7 | 29.7 | 29.9 |
| 2 | 29.3 | 32.2 | 31.8 | 30.8 | 29 | 30.8 | 29 | 28.6 | 29.8 | 29.9 |
| 3 | 29.1 | 32.1 | 31.9 | 30.7 | 29.1 | 30.9 | 29.1 | 28.7 | 29.6 | 29.8 |
| 4 | 29.2 | 32.1 | 31.7 | 30.9 | 29 | 30.9 | 28.9 | 28.6 | 29.7 | 29.9 |
| 5 | 29.6 | 32.2 | 31.8 | 30.6 | 29.1 | 30.8 | 29 | 28.7 | 29.7 | 30 |
| 6 | 29.5 | 31.9 | 31.7 | 30.7 | 29 | 30.9 | 29 | 28.6 | 29.8 | 29.9 |
| 7 | 29.7 | 32.1 | 31.6 | 30.7 | 29.1 | 31 | 29.1 | 28.7 | 29.6 | 29.8 |
| 8 | 29.9 | 32.1 | 31.7 | 30.7 | 29 | 31 | 29 | 28.6 | 29.7 | 29.8 |
| 9 | 30.1 | 32 | 31.7 | 30.8 | 29 | 30.8 | 29.1 | 28.6 | 29.8 | 29.8 |
| 10 | 28.7 | 31.9 | 31.8 | 30.7 | 29.1 | 31 | 29 | 28.7 | 29.8 | 29.7 |
| 11 | 29.1 | 32 | 31.8 | 30.7 | 29 | 30.9 | 29 | 28.7 | 29.7 | 29.8 |
| 12 | 28.9 | 32.1 | 31.6 | 30.7 | 29.1 | 30.8 | 29 | 28.7 | 29.7 | 29.8 |
| 13 | 29.0 | 32.1 | 31.9 | 30.8 | 29.2 | 30.9 | 29 | 28.7 | 29.7 | 29.8 |
| 14 | 29.0 | 31.9 | 31.7 | 30.7 | 29 | 30.9 | 28.9 | 28.6 | 29.6 | 29.8 |
| 15 | 29.5 | 32.2 | 31.8 | 30.8 | 29 | 30.9 | 29 | 28.6 | 29.6 | 29.9 |
| 16 | 29.3 | 32.1 | 31.7 | 30.7 | 29 | 30.8 | 29.1 | 28.7 | 29.6 | 29.8 |
| 17 | 28.9 | 32.1 | 31.8 | 30.6 | 29 | 31 | 29.1 | 28.8 | 29.6 | 29.9 |
| 18 | 29.0 | 32.1 | 31.7 | 30.6 | 29 | 31 | 29.1 | 28.7 | 29.7 | 29.8 |
| 19 | 29.1 | 32 | 31.9 | 30.7 | 29.1 | 30.8 | 29.1 | 28.6 | 29.7 | 29.8 |
| 20 | 28.9 | 32 | 31.7 | 30.7 | 29.1 | 31 | 29 | 28.6 | 29.7 | 29.8 |
| 21 | 29.1 | 32 | 31.6 | 30.7 | 29 | 30.9 | 29.1 | 28.7 | 29.8 | 29.9 |
| 22 | 28.9 | 32.1 | 31.7 | 30.7 | 29.1 | 30.8 | 29 | 28.8 | 29.6 | 29.9 |
| 23 | 29.1 | 32.1 | 31.7 | 30.7 | 29 | 30.9 | 29 | 28.7 | 29.7 | 29.8 |
| 24 | 29.3 | 31.9 | 31.7 | 30.8 | 29.2 | 30.9 | 29 | 28.7 | 29.7 | 29.9 |
| 25 | 29.2 | 32.1 | 31.8 | 30.8 | 29 | 30.9 | 29 | 28.7 | 29.8 | 29.8 |
| 26 | 28.9 | 32.1 | 31.8 | 30.8 | 29 | 30.8 | 29.1 | 28.8 | 29.8 | 30 |
| 27 | 29.1 | 32.1 | 31.7 | 30.6 | 29.1 | 31 | 29.1 | 28.7 | 29.7 | 30 |
| 28 | 29.1 | 32 | 31.7 | 30.7 | 29 | 30.9 | 29 | 28.7 | 29.8 | 29.9 |
| 29 | 30.1 | 32 | 31.7 | 30.6 | 29 | 31 | 29 | 28.7 | 29.7 | 29.8 |
| 30 | 29.1 | 32 | 31.6 | 30.8 | 29.1 | 30.9 | 29.1 | 28.7 | 29.8 | 29.9 |
| 31 | 28.9 | 32.1 | 31.7 | 30.8 | 29 | 30.9 | 28.9 | 28.8 | 29.7 | 29.9 |
| 31 | 29.1 | 32.1 | 31.6 | 30.8 | 29.1 | 31 | 29 | 28.8 | 29.5 | 29.9 |
| 33 | 29.3 | 31.9 | 31.8 | 30.7 | 29.1 | 30.9 | 29 | 28.6 | 29.8 | 29.8 |
| 34 | 29.0 | 32.1 | 31.8 | 30.7 | 29 | 31 | 29.1 | 28.7 | 29.8 | 29.8 |
| 35 | 29 | 32 | 31.7 | 30.7 | 29.1 | 30.9 | 29 | 28.7 | 29.8 | 29.8 |
| 36 | 29.1 | 31.3 | 31.7 | 30.8 | 29 | 31 | 29.1 | 28.6 | 29.6 | 29.8 |
| 37 | 29.2 | 32 | 31.7 | 30.7 | 29.1 | 30.9 | 29.1 | 28.6 | 29.5 | 29.8 |
| 38 | 29.2 | 32.1 | 31.8 | 30.7 | 29 | 31 | 28.9 | 28.6 | 29.6 | 29.8 |
| 39 | 29.1 | 32 | 31.8 | 30.7 | 29.1 | 31 | 28.9 | 28.7 | 29.6 | 29.9 |
| 40 | 28.9 | 32.1 | 31.8 | 30.8 | 29.1 | 30.9 | 28.9 | 28.7 | 29.5 | 30 |
| 41 | 29.1 | 32 | 31.7 | 30.8 | 29.1 | 31 | 29 | 28.7 | 29.7 | 29.9 |
| 42 | 29 | 32 | 31.7 | 30.8 | 29 | 31 | 29.1 | 28.6 | 29.7 | 29.8 |
| 43 | 29.1 | 32 | 31.7 | 30.7 | 29.1 | 30 | 29.1 | 28.7 | 29.7 | 29.9 |
| 44 | 29.2 | 32.1 | 31.7 | 30.7 | 29.1 | 30.9 | 29 | 28.7 | 29.7 | 30 |
| 45 | 29 | 32 | 31.7 | 30.6 | 29 | 30.9 | 29.1 | 28.7 | 29.7 | 29.8 |
| 46 | 28.9 | 32 | 31.7 | 30.7 | 29 | 30.9 | 29 | 28.8 | 29.7 | 29.9 |
| 47 | 29.2 | 32.1 | 31.7 | 30.7 | 29.1 | 30.8 | 29.1 | 28.7 | 29.8 | 29.8 |
| 48 | 29.1 | 32 | 31.7 | 30.8 | 29 | 31 | 29.3 | 28.8 | 29.8 | 29.8 |
| 49 | 29 | 32 | 31.7 | 30.7 | 29.1 | 30.8 | 29 | 28.6 | 29.5 | 29.9 |
| 50 | 29.2 | 32.1 | 31.8 | 30.8 | 29 | 30.9 | 28.9 | 28.7 | 29.6 | 29.8 |

Tabel 7. The data of egg weight increase (%) of *Hemibagrus wyckii*

| **No** | **Number of fish** | | | | | | | | | |
| --- | --- | --- | --- | --- | --- | --- | --- | --- | --- | --- |
|  | **1** | **2** | **3** | **4** | **5** | **6** | **7** | **8** | **9** | **10** |
| 1 | 20 | 30 | 20 | 30 | 20 | 20 | 20 | 30 | 20 | 30 |
| 2 | 30 | 30 | 20 | 20 | 20 | 20 | 20 | 20 | 20 | 20 |
| 3 | 30 | 30 | 30 | 20 | 20 | 20 | 20 | 20 | 20 | 20 |
| 4 | 20 | 30 | 20 | 30 | 20 | 20 | 20 | 20 | 20 | 20 |
| 5 | 30 | 30 | 20 | 20 | 20 | 20 | 20 | 20 | 20 | 20 |
| 6 | 20 | 20 | 20 | 30 | 20 | 30 | 20 | 20 | 30 | 30 |
| 7 | 20 | 30 | 20 | 20 | 20 | 20 | 20 | 30 | 20 | 20 |
| 8 | 20 | 20 | 20 | 20 | 20 | 30 | 20 | 20 | 20 | 30 |
| 9 | 20 | 20 | 20 | 20 | 20 | 20 | 20 | 20 | 20 | 30 |
| 10 | 20 | 20 | 20 | 20 | 20 | 20 | 20 | 20 | 20 | 20 |
| 11 | 20 | 20 | 30 | 20 | 20 | 30 | 20 | 30 | 20 | 20 |
| 12 | 20 | 20 | 20 | 20 | 20 | 20 | 20 | 20 | 20 | 20 |
| 13 | 20 | 30 | 30 | 20 | 30 | 20 | 20 | 30 | 20 | 20 |
| 14 | 10 | 20 | 20 | 20 | 20 | 20 | 20 | 20 | 20 | 20 |
| 15 | 30 | 30 | 30 | 20 | 20 | 20 | 20 | 20 | 20 | 20 |
| 16 | 20 | 20 | 20 | 20 | 20 | 20 | 30 | 20 | 20 | 30 |
| 17 | 20 | 20 | 20 | 20 | 20 | 20 | 20 | 20 | 20 | 30 |
| 18 | 20 | 20 | 20 | 20 | 20 | 30 | 30 | 20 | 20 | 10 |
| 19 | 20 | 20 | 30 | 20 | 20 | 20 | 30 | 20 | 20 | 20 |
| 20 | 20 | 20 | 20 | 20 | 20 | 20 | 20 | 20 | 20 | 20 |
| 21 | 20 | 20 | 20 | 20 | 20 | 20 | 20 | 20 | 30 | 30 |
| 22 | 30 | 20 | 20 | 20 | 20 | 20 | 30 | 20 | 20 | 20 |
| 23 | 20 | 20 | 20 | 20 | 20 | 20 | 30 | 30 | 20 | 20 |
| 24 | 20 | 20 | 20 | 20 | 30 | 20 | 30 | 20 | 20 | 20 |
| 25 | 20 | 30 | 20 | 20 | 30 | 20 | 20 | 20 | 20 | 20 |
| 26 | 20 | 20 | 20 | 20 | 20 | 20 | 30 | 30 | 20 | 30 |
| 27 | 20 | 20 | 20 | 10 | 20 | 20 | 20 | 30 | 20 | 30 |
| 28 | 20 | 20 | 20 | 20 | 20 | 20 | 20 | 20 | 30 | 20 |
| 29 | 30 | 20 | 20 | 10 | 20 | 20 | 20 | 20 | 20 | 20 |
| 30 | 20 | 20 | 10 | 20 | 20 | 20 | 30 | 30 | 30 | 20 |
| 31 | 20 | 20 | 20 | 20 | 20 | 20 | 20 | 20 | 20 | 20 |
| 32 | 20 | 20 | 20 | 20 | 20 | 20 | 30 | 30 | 0 | 30 |
| 33 | 20 | 20 | 20 | 20 | 30 | 30 | 20 | 20 | 20 | 20 |
| 34 | 30 | 20 | 20 | 20 | 20 | 30 | 20 | 30 | 20 | 30 |
| 35 | 20 | 20 | 20 | 20 | 20 | 20 | 20 | 20 | 20 | 20 |
| 36 | 20 | 20 | 20 | 20 | 20 | 20 | 30 | 20 | 20 | 20 |
| 37 | 20 | 20 | 20 | 20 | 20 | 20 | 20 | 20 | 20 | 20 |
| 38 | 20 | 20 | 20 | 20 | 20 | 20 | 20 | 20 | 20 | 20 |
| 39 | 20 | 20 | 20 | 20 | 20 | 20 | 20 | 20 | 20 | 20 |
| 40 | 20 | 20 | 20 | 20 | 20 | 20 | 20 | 30 | 20 | 30 |
| 41 | 20 | 20 | 20 | 20 | 20 | 30 | 20 | 30 | 20 | 20 |
| 42 | 20 | 20 | 20 | 20 | 20 | 30 | 30 | 20 | 20 | 20 |
| 43 | 20 | 20 | 20 | 20 | 20 | 20 | 20 | 20 | 20 | 30 |
| 44 | 20 | 20 | 10 | 20 | 20 | 20 | 30 | 30 | 20 | 30 |
| 45 | 20 | 20 | 20 | 20 | 20 | 20 | 20 | 30 | 20 | 20 |
| 46 | 20 | 20 | 20 | 20 | 20 | 20 | 20 | 30 | 20 | 20 |
| 47 | 20 | 20 | 30 | 20 | 20 | 20 | 20 | 30 | 20 | 20 |
| 48 | 20 | 20 | 20 | 20 | 20 | 20 | 30 | 30 | 20 | 20 |
| 49 | 20 | 20 | 20 | 20 | 20 | 20 | 20 | 20 | 20 | 20 |
| 50 | 30 | 20 | 20 | 20 | 20 | 20 | 20 | 30 | 20 | 20 |

Tabel 8. The data of Fertilization rate (%) of *Hemibagrus wyckii*

|  | **Number of Fish** | | | | | | | | | |
| --- | --- | --- | --- | --- | --- | --- | --- | --- | --- | --- |
| **Fertilization rate (%)** | **1** | **2** | **3** | **4** | **5** | **6** | **7** | **8** | **9** | **10** |
| 1 | 68 | 70 | 62 | 66 | 54 | 54 | 62 | 62 | 62 | 64 |
| 2 | 66 | 65 | 60 | 68 | 54 | 56 | 60 | 60 | 60 | 60 |
| 3 | 72 | 64 | 60 | 64 | 52 | 54 | 59 | 63 | 61 | 58 |

Tabel 9. The data of Hatching rate (%) of *Hemibagrus wyckii*

|  |  | **Number of fish** | | | | | | | | |
| --- | --- | --- | --- | --- | --- | --- | --- | --- | --- | --- |
| **Hatching rate (%)1** | **1** | **2** | **3** | **4** | **5** | **6** | **7** | **8** | **9** | **10** |
| 1 | 48 | 44 | 41 | 46 | 44 | 42 | 40 | 40 | 40 | 40 |
| 2 | 46 | 48 | 42 | 48 | 44 | 44 | 42 | 44 | 38 | 42 |
| 3 | 52 | 44 | 39 | 44 | 42 | 48 | 38 | 40 | 44 | 36 |

Tabel 10. The data of Hatching weight (mg) of *Hemibagrus wyckii*

|  | **Number of Fish** | | | | | | | | | |
| --- | --- | --- | --- | --- | --- | --- | --- | --- | --- | --- |
| No | 1 | 2 | 3 | 4 | 5 | 6 | 7 | 8 | 9 | 10 |
| 1 | 28 | 33 | 30 | 30 | 29 | 28 | 30 | 31 | 30 | 29 |
| 2 | 29 | 33 | 31 | 31 | 29 | 29 | 30 | 30 | 29 | 30 |
| 3 | 28 | 33 | 33 | 30 | 29 | 28 | 30 | 30 | 29 | 29 |
| 4 | 28 | 32 | 31 | 31 | 30 | 29 | 31 | 30 | 30 | 28 |
| 5 | 29 | 32 | 31 | 31 | 31 | 29 | 31 | 30 | 30 | 28 |
| 6 | 29 | 32 | 31 | 31 | 30 | 28 | 31 | 31 | 30 | 28 |
| 7 | 28 | 33 | 32 | 32 | 29 | 29 | 32 | 30 | 30 | 28 |
| 8 | 29 | 32 | 33 | 30 | 28 | 28 | 30 | 30 | 30 | 29 |
| 9 | 29 | 32 | 32 | 31 | 29 | 29 | 29 | 31 | 30 | 30 |
| 10 | 29 | 32 | 32 | 32 | 29 | 29 | 32 | 31 | 31 | 29 |
| 11 | 29 | 32 | 32 | 31 | 30 | 28 | 31 | 31 | 31 | 29 |
| 12 | 29 | 32 | 31 | 31 | 32 | 32 | 31 | 31 | 30 | 31 |
| 13 | 28 | 33 | 31 | 31 | 29 | 29 | 31 | 30 | 30 | 30 |
| 14 | 28 | 33 | 31 | 31 | 29 | 29 | 31 | 31 | 30 | 28 |
| 15 | 29 | 33 | 32 | 31 | 28 | 28 | 31 | 31 | 31 | 30 |
| 16 | 29 | 33 | 31 | 29 | 29 | 29 | 29 | 31 | 31 | 28 |
| 17 | 28 | 33 | 33 | 30 | 29 | 29 | 30 | 31 | 31 | 30 |
| 18 | 29 | 33 | 31 | 31 | 29 | 29 | 31 | 31 | 31 | 30 |
| 19 | 29 | 33 | 30 | 30 | 30 | 28 | 30 | 30 | 30 | 29 |
| 20 | 29 | 32 | 31 | 30 | 29 | 29 | 30 | 30 | 30 | 30 |
| 21 | 29 | 32 | 31 | 30 | 30 | 30 | 30 | 30 | 30 | 28 |
| 22 | 29 | 32 | 30 | 30 | 29 | 29 | 30 | 30 | 30 | 31 |
| 23 | 29 | 32 | 30 | 30 | 28 | 28 | 30 | 30 | 30 | 28 |
| 24 | 29 | 32 | 30 | 30 | 28 | 28 | 30 | 29 | 29 | 28 |
| 25 | 28 | 32 | 30 | 30 | 28 | 28 | 30 | 29 | 29 | 28 |
| 26 | 28 | 32 | 30 | 30 | 28 | 28 | 30 | 31 | 31 | 28 |
| 27 | 28 | 32 | 30 | 29 | 29 | 29 | 29 | 29 | 29 | 29 |
| 28 | 28 | 33 | 30 | 30 | 30 | 30 | 30 | 31 | 31 | 28 |
| 29 | 29 | 33 | 31 | 31 | 29 | 29 | 31 | 31 | 31 | 28 |
| 30 | 28 | 32 | 30 | 30 | 29 | 29 | 30 | 31 | 31 | 28 |
| 31 | 29 | 32 | 31 | 29 | 31 | 31 | 29 | 31 | 31 | 29 |
| 32 | 29 | 32 | 31 | 31 | 30 | 30 | 31 | 31 | 31 | 28 |
| 33 | 29 | 32 | 32 | 30 | 28 | 28 | 30 | 31 | 31 | 29 |
| 34 | 29 | 32 | 32 | 32 | 30 | 30 | 32 | 31 | 31 | 28 |
| 35 | 28 | 31 | 31 | 31 | 30 | 30 | 31 | 31 | 31 | 29 |
| 36 | 29 | 31 | 32 | 32 | 30 | 30 | 30 | 31 | 31 | 29 |
| 37 | 29 | 31 | 31 | 29 | 30 | 30 | 29 | 31 | 31 | 28 |
| 38 | 28 | 32 | 31 | 30 | 29 | 29 | 30 | 31 | 31 | 29 |
| 39 | 28 | 33 | 31 | 30 | 30 | 30 | 30 | 30 | 30 | 28 |
| 40 | 29 | 32 | 31 | 29 | 30 | 28 | 29 | 31 | 31 | 29 |
| 41 | 28 | 32 | 33 | 30 | 31 | 31 | 29 | 32 | 32 | 29 |
| 42 | 29 | 32 | 31 | 29 | 30 | 28 | 28 | 31 | 31 | 28 |
| 43 | 28 | 32 | 31 | 29 | 31 | 28 | 29 | 31 | 31 | 30 |
| 44 | 29 | 32 | 31 | 29 | 32 | 28 | 28 | 30 | 30 | 29 |
| 45 | 29 | 32 | 32 | 29 | 31 | 28 | 28 | 31 | 31 | 29 |
| 46 | 29 | 32 | 33 | 29 | 29 | 29 | 28 | 32 | 32 | 28 |
| 47 | 28 | 31 | 32 | 29 | 29 | 28 | 29 | 31 | 31 | 29 |
| 48 | 29 | 31 | 31 | 29 | 30 | 28 | 29 | 29 | 29 | 29 |
| 49 | 29 | 32 | 31 | 31 | 31 | 28 | 31 | 30 | 30 | 29 |
| 50 | 29 | 32 | 32 | 29 | 29 | 29 | 29 | 29 | 29 | 28 |

Tabel 11. Male size, gonada weight and semen of *Hemibagrus wyckii*

| **Number fish** | **Fish weight (g)** | **Fish length (cm)** | **Gonada weight (g)** | **Gonadosomatic index (%)** | **Semen volume (mL)** | **Semen pH** |
| --- | --- | --- | --- | --- | --- | --- |
| 1 | 2099 | 56 | 32.95 | 1.57 | 1.2 | 7.5 |
| 2 | 1404 | 52.5 | 26.9 | 2.83 | 0.8 | 7.6 |
| 3 | 1932 | 56.2 | 30.81 | 2.63 | 0.8 | 7.7 |
| 4 | 2498 | 65.2 | 33.71 | 1.06 | 0.8 | 7.5 |
| 5 | 1456 | 45.9 | 24.96 | 2.81 | 0.7 | 7.6 |
| 6 | 1714 | 58.3 | 28.85 | 1.1 | 0.6 | 7.5 |
| 7 | 1819 | 54.8 | 26.87 | 1.59 | 0.7 | 7.4 |
| 8 | 2127 | 64.3 | 33.86 | 1.31 | 0.6 | 7.3 |
| 9 | 1237 | 43.7 | 24.98 | 2.02 | 0.9 | 7.4 |
| 10 | 1405 | 48.3 | 28.95 | 2.62 | 1.1 | 7.5 |

Tabel 12. Sperm concentration (10^9^/ml) of *Hemibagrus wyckii*

| **Replicate** | **Number of fish** | | | | | | | | | |
| --- | --- | --- | --- | --- | --- | --- | --- | --- | --- | --- |
|  | **1** | **2** | **3** | **4** | **5** | **6** | **7** | **8** | **9** | **10** |
| 1 | 3.9 | 3.8 | 3.7 | 3.9 | 3.5 | 3.6 | 3.5 | 3.6 | 3.7 | 3.6 |
| 2 | 3.8 | 4.1 | 3.9 | 4 | 3.4 | 3.7 | 3.4 | 3.4 | 3.9 | 3.8 |
| 3 | 4.2 | 3.7 | 3.6 | 3.8 | 3.6 | 3.5 | 3.4 | 3.8 | 3.6 | 3.4 |

Tabel 13. Sperm Motility (%) of *Hemibagrus wyckii*

|  | **Number of fish** | | | | | | | | | |
| --- | --- | --- | --- | --- | --- | --- | --- | --- | --- | --- |
| **Replicate** | **1** | **2** | **3** | **4** | **5** | **6** | **7** | **8** | **9** | **10** |
| 1 | 72 | 72.5 | 71.5 | 74 | 74 | 75 | 71 | 73.5 | 73 | 75 |
| 2 | 70 | 73 | 73 | 72.5 | 73.5 | 74 | 72 | 72.5 | 74 | 76 |
| 3 | 69 | 71.5 | 70.5 | 73 | 72 | 75 | 72 | 71.5 | 74 | 74 |

Tabel 14. Duration motility (sec) of *Hemibagrus wyckii*

|  | **Number of fish** | | | | | | | | | |
| --- | --- | --- | --- | --- | --- | --- | --- | --- | --- | --- |
| **Replicate** | **1** | **2** | **3** | **4** | **5** | **6** | **7** | **8** | **9** | **10** |
| 1 | 40 | 47 | 43 | 48 | 52 | 47 | 43 | 54 | 52 | 54 |
| 2 | 42 | 43 | 42 | 50 | 48 | 45 | 46 | 54 | 53 | 53 |
| 3 | 38 | 45 | 46 | 46 | 50 | 43 | 43 | 53 | 56 | 55 |
