# Supplementary material for: Reproductive performance of asian catfish ( Hemibagrus wyckii Bleeker, 1858), a candidate species for aquaculture
Source: F1000Res. 2018 Sep 3;7:683. Originally published 2018 May 31. [Version 2] doi: 10.12688/f1000research.14746.2 (PMC6107977; doi:10.12688/f1000research.14746.2)
Supplement: Peer review history

# Peer review: Referee response for version 2

**DOI:** 10.5256/f1000research.17617.r37837

**Published:** 2019-01-10

**Authors:** Zainal Abidin Muchlisin

**Affiliations:** 1 Faculty of Marine and Fisheries, Syiah Kuala University, Banda Aceh, Indonesia

The authors have made corrections as suggested

I have read this submission. I believe that I have an appropriate level of expertise to confirm that it is of an acceptable scientific standard.

# Peer review: Referee response for version 1

**DOI:** 10.5256/f1000research.16043.r37278

**Published:** 2018-08-22

**Authors:** Rudy Agung Nugroho

**Affiliations:** 1 Department of Biology, Mulawarman University, Samarinda, Indonesia

**Title**

 The title should be revised: delete “a candidate species for aquaculture”. It needs more parameters (Growth, feed aspect, etc.)  to convey that this species can be a candidate for aquaculture.

**Abstract**

 In methods: the author stated that “Egg size (50 eggs for each fish) was measured using an Olympus microscope (CX40)”. This statement should be revised, since the microscope CX40 itself cannot be used to measure the egg size. It must be a micrometer within a microscope that has been used to measure the egg size, or the author has another technique to measure the egg size. This method should be also clearly stated in the methods section.

**Introduction**

 The introduction should be extended by providing more information about Asian catfish and their biological, physiological, and ecological aspects.

**Methods**

**Ethical consideration**: Please revise, it is seems there is a typo:

 "[...]the Government of the Republic of Indonesia does not require licences to be obtain to capture and rear this species, hense no licences are applicable to this study." This sentence should be rewritten: "[...] the Government of the Republic of Indonesia does not require licenses to obtain, capture and rear this species. Hence, no licenses are applicable to this study."

 "In the end of the experiment the *H. wyckii* still in good condition until return back to the pond." The word pond should be changed to river, because the fish is returned back to the original (River not pond).

**Rearing and selection of breeders: **"Then, the broodstock of *H. wyckii* had been adapted and grown to maturation under the farm conditions”. The word grown should be changed to reared, because this experiment is not about growth.

**Water quality**:  “Alkalinity and hardness levels of the water were measured in each replicate according to standard procedures”.  It is not clear what “each replicate” is referring to. Does it mean that the measurement was done in a few replications? Why only alkalinity and hardness?

**Results**

 Results are well presented.

**Discussion**

 The discussion can be extended by explaining the relationship between the results of water quality and the biological reproduction aspect, since the purpose of this study is under culture condition.

**Conclusion**

 Needs slight revision based on all aspects of the findings.

I have read this submission. I believe that I have an appropriate level of expertise to confirm that it is of an acceptable scientific standard.

# Peer review: Referee response for version 1

**DOI:** 10.5256/f1000research.16043.r34564

**Published:** 2018-06-11

**Authors:** Zainal Abidin Muchlisin

**Affiliations:** 1 Faculty of Marine and Fisheries, Syiah Kuala University, Banda Aceh, Indonesia

**Title:**

1. I think the title should be revised as "Reproductive biology and breeding of the Asian catfish ( *Hemibagrus wyckii*) after domestication process.
2. In the title: (Hemibagris wyckii Bagridae) is missed understanding, the correct written should be *Hemibagrus wyckii*: Bagridae), because Bagridae is a Family level, or you can write in as ( *Hemigarus wyckii *Bleeker, 1858)

**Abstract:**

 Background: ..... I think "most" should be deleted because sound so emotional or hyperbolic, just say ".... is one of the important economic fish species...."

 Methods: Please mention where the brood fish is come from?

 Conclusion: The conclusions is out of the context. You have to make conclusion  just based on the data (findings), forget for candidate species for aquaculture, because, there was no detail discussion about this issue, so just focus on your present findings.

 Keywords: I suggest don't use similar words which already exist in the title.

**Introduction:**

 I think the state of the arts about the asian catfish is still shallow or unclear, so please provide more information about the previous studies on this species related to bio ecology, feeding, aquaculture etc.

 Please add some information what is the advantages of the species compared to other freshwater species?

**Methods:**

 Please cite these references to enhance your methods:

- Muchlisin *et al*., 2010 ^1^
- Muchlisin *et al., *2011 ^2^

**Results:**

 The results have been explained in several short paragraphs, please extend the paragraph by combining paragraph 1, 2 in one paragraph and paragraph 3 and 4 in one paragraph.

**Discussion:**

 There was no comprehensive discussion to justify why this species is potential for aquaculture.  The justification is not only based on biological aspects but also on economic consideration (see Muchlisin, 2013 ^3^).

 Unfortunately, there was no economic evaluation performed.Therefore, I suggest to focus only on the finding as already presented.

**Conclusion:**

 The conclusion has to be revised. Conclusion is based on the finding, not assumption or interpretation.

I have read this submission. I believe that I have an appropriate level of expertise to confirm that it is of an acceptable scientific standard.

## References

[1] MuchlisinZAMusmanMAzizahMN: Spawning seasons of Rasbora tawarensis (Pisces: Cyprinidae) in Lake Laut Tawar, Aceh province, Indonesia. *Reprod Biol Endocrinol*.2010;8: 10.1186/1477-7827-8-49 49 10.1186/1477-7827-8-49 20482777PMC2887878

[2] MuchlisinZAMusmanMFadliNNor Siti-AzizahN: Fecundity and spawning frequency of Rasbora tawarensis (Pisces: Cyprinidae) an endemic species from Lake Laut Tawar, Aceh, Indonesia. *AACL Bioflux*.2011;4(3) :

[3] MuchlisinZA: Potency of freshwater fishes in Aceh waters as a basis for aquaculture development program. *Indonesian Journal of Ichthyology*.2013;13(1) :
